# Data showing regional differences in rat brain monoaminergic function

**DOI:** 10.1016/j.dib.2019.104814

**Published:** 2019-11-15

**Authors:** A.J.D. Nelson, H.J. Cassaday

**Affiliations:** a School of Psychology, University of Nottingham, UK; b School of Psychology, Cardiff University, UK

**Keywords:** Monoamines, Rat, HPLC-ECD, Prefrontal cortex, Nucleus accumbens

## Abstract

Chemical neurotransmitters (such as dopamine) modulate cognitive function via ascending projections to various cortical and sub-cortical brain regions. This report describes and links to a relatively large dataset (up to *N* = 112) compiled from control (untreated) brain samples taken during a series of experimental *in vivo* studies. The dataset is freely available, to explore the normal interrelationships between levels of neurotransmitter (e.g., dopamine, serotonin), across brain regions implicated in both normal reward and drug addiction, as well as in disorders such as schizophrenia (e.g., nucleus accumbens, prefrontal cortex). Most experimental studies run with a relatively small control group, so there is a lack of baseline data on the expected levels of neurotransmitters and their metabolites in different brain regions. Accordingly, the available dataset has been compiled from a number of studies run in the same laboratory, and using closely similar behavioural procedures, sampling selected brain regions of *a priori* interest. These collated data can be used to explore differences in the distribution of the monoamines and their metabolites, patterns of neurotransmitter intercorrelations, both between and within different brain structures and including some consideration of laterality effects.

## Abbreviations

NAc: nucleus accumbens
PL: prelimbic cortex
IL: infralimbic cortex
mPFC: medial prefrontal cortex
OFC: orbitofrontal cortex
DLS: dorsolateral striatum (caudate putamen)
Amyg: amygdala
6-OHDA: 6-hydroxydopamine
5,7-DHT: 5,7-dihydroxytryptamine
MDMA: 3,4-methylenedioxymethamphetamine
DA: dopamine
NA: noradrenalin
5-HT: serotonin
DOPAC: 3,4-dihydroxyphenylacetic acid
5-HIAA: 5-hydroxyindoleacetic acid
HVA: homovanillic acid
HPLC-ECD: high performance liquid chromatography with electrochemical detection

**Table:** Specifications Table

Subject	Neuroscience
Specific subject area	Regional differences in rat brain monoaminergic function
Type of data	Table (data origins) Figures (averaged data from preliminary analyses in SPSS) Excel (raw Dataset 1 in Supplementary Material 1) SPSS (raw Dataset 2 in Supplementary Material 2)
How data were acquired	High performance liquid chromatography with electrochemical detection using a glassy carbon electrode flow cell (VT-03 Antec) with an ISAAC reference electrode. Samples were subsequently analysed using the Alexys software data system.
Data format	Raw Analysed
Parameters for data collection	The data are compiled from control (untreated) brain samples taken during a series of experimental *in vivo* studies. Tissue samples were taken only from a limited number of brain regions of *a priori* interest.
Description of data collection	These studies followed a standard methodology for tissue sampling and HPLC-ECD. In each case, the rats were humanely killed by dislocation of the neck and decapitated; the brains were removed rapidly and dissected on a cold tray. Tissue samples were then taken using a micro-punch procedure from 2 mm coronal brain sections. Brain regions were identified using *The Rat Brain in Stereotaxic Coordinates* [1], using 0.84 and 1.6mm punches (depending on the size of the region to be sampled).
Data source location	Nottingham UK
Data accessibility	The data are hosted with the article.

**Table:** 

**Value of the Data** • The dataset contributes a large sample of baseline data (up to *N* = 112) to compare with other assays of neurotransmitters and their metabolites in selected brain regions, including data by cerebral hemisphere (laterality) where available. • Neuroscientists, neuropharmacologists and statisticians can benefit from this dataset, which allows investigation of intercorrelations between monoaminergic parameters in cortical and sub-cortical structures. • The dataset allows interrogation, both for exploratory approaches and to test specific hypotheses as to how functional interconnectivity (Fig. 1) is reflected in neurotransmitter function. **Fig. 1:** 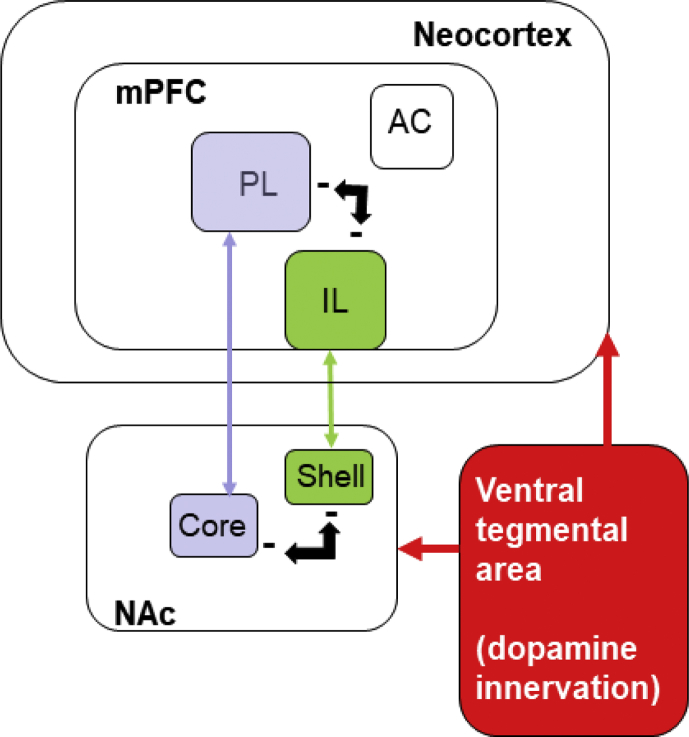 Medial prefrontal cortex (mPFC) and nucleus accumbens (NAc) interconnectivity. Local inhibitory interactions between prelimbic (PL) and infralimbic (IL) areas of mPFC and between core and shell sub-regions of NAc are denoted by **-**. • The dataset can be used to provide further insights into other functional interrelationships between neurotransmitter systems in the regions examined. • This dataset can be used to inform the development of future experiments to examine functional interconnectivity, in particular disconnection studies in the case of differences by cerebral laterality. • The additional value of the pooled dataset provided in the present resource is the derived from the sample size and provision of data by cerebral laterality (where available).

### 1 Data

**Fig. 2:**
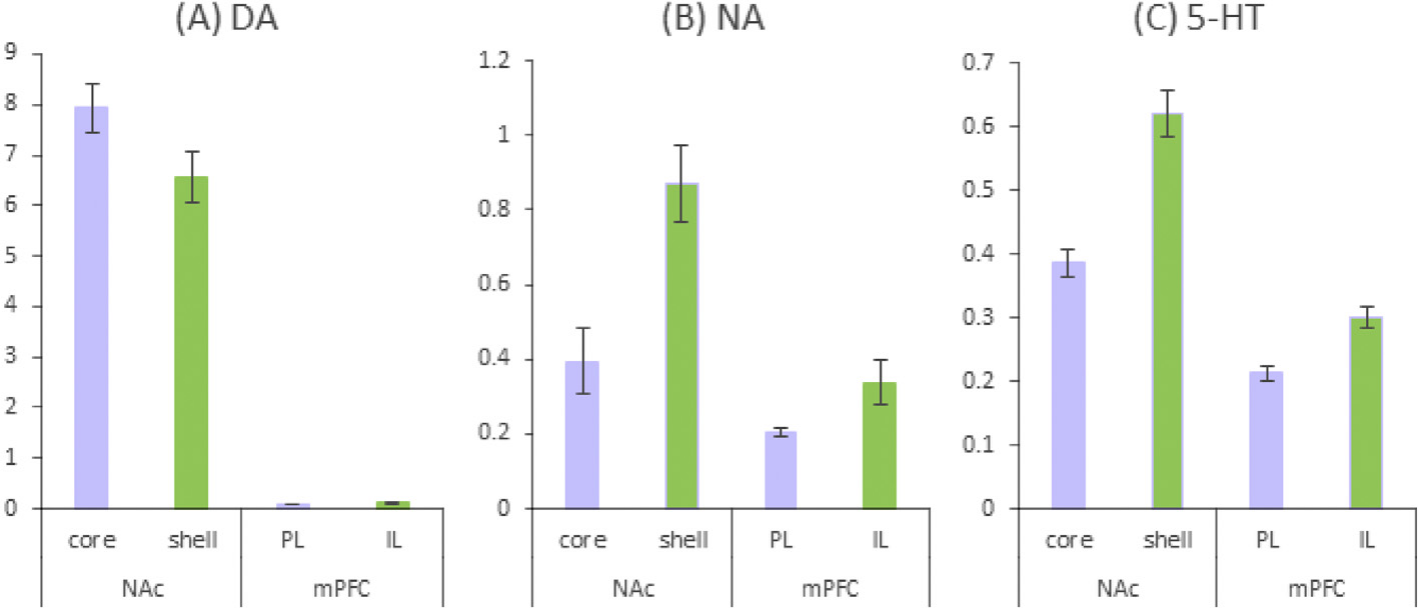
Summary data from analysis in SPSS of the raw data provided in Dataset 2. Mean levels of (A) dopamine (DA) *N* = 92, (B) noradrenalin *N* = 56 and (C) serotonin (5-HT) *N* = 92 expressed as pmoles per μg of protein content for core and shell nucleus accumbens (NAc) and prelimbic (PL) and infralimbic (IL) medial prefrontal cortex (mPFC).

**Fig. 3:**
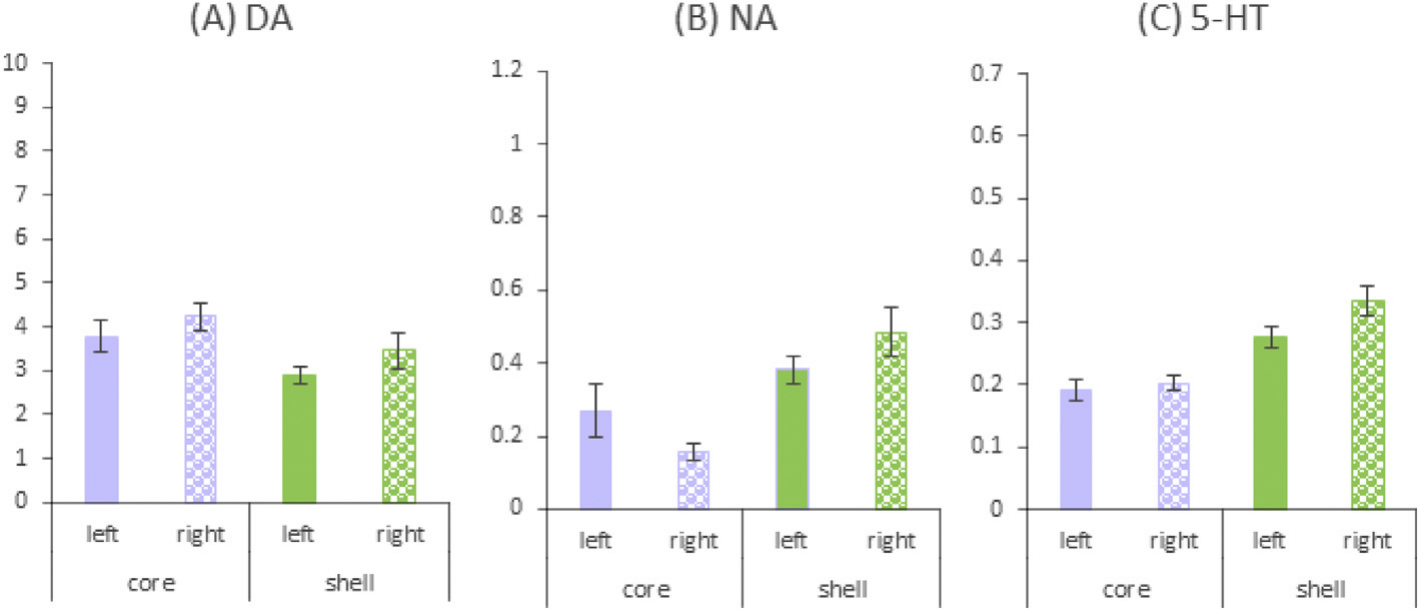
Summary data from analysis in SPSS of the raw data provided in Dataset 2. Mean levels of (A) dopamine (DA) *N* = 98, (B) noradrenalin *N* = 62 and (C) serotonin (5-HT) *N* = 98 expressed as pmoles per μg of protein content for core and shell nucleus accumbens (NAc) by left and right hemisphere.

The dataset provided with this article contains raw HPLC-ECD measures in two formats (Dataset 1 in Supplementary Material 1; Dataset 2 in Supplementary Material 2), together with some summary data (Fig. 2, Fig. 3). The dataset was compiled from control (untreated) brain samples taken during a series of experimental *in vivo* studies (Table 1). Tissue samples were taken only from a limited number of brain regions of *a priori* interest.

**Table 1:** Data origins by brain regions available and publication reference.

Studies pooled	Control sample *N*	Brain regions available				Publication reference
NAc 6-OHDA 1&2	36	NAc core vs shell	DLS	PL, IL		Journal of Psychopharmacology, 25, 1649–1660 [5]
NAc 6-OHDA 3	23	NAc core vs shell	DLS	PL, IL		Pharmacology, Biochemistry and Behavior, 98, 1–7 [4]
PL/IL 6-OHDA 1	20	NAc core vs shell	DLS	PL, IL, OFC	Amyg	Neuroscience, 170, 99–106 [3]
NAc 5,7-DHT 1	21	NAc core vs shell	DLS	PL, IL	Amyg	International Journal of Neuropsychopharmacology, 15, 485–496 [6]
MDMA – systemic	12	NAc		mPFC	Amyg	Neuropharmacology, 67, 331–336 [7]

This dataset can be used for exploratory approaches and to test specific hypotheses as to how functional interconnectivity is reflected in neurotransmitter function [2]. The dataset can further be used to identify laterality differences in neurotransmitter function. These are of interest in their own right and can in principle be taken into account in statistical models of functional interconnectivity mediated by the monoamines. When samples are taken to verify the effectiveness of an experimental intervention such as the injection of a neurotoxin, the convention is to pool the samples from the right and left hemispheres prior to analysis and this is the approach that we have previously adopted, to assess the effects of dopaminergic depletion using 6-OHDA [[3], [4], [5]], serotonergic depletion using 5,7-DHT [6], and systemic MDMA [7].

Table 1 shows how the present dataset derives from the previously published studies (labelled by the intervention in use in the experimental group and any applicable replication number), together with the maximum sample sizes and brain regions available.

#### 1.1 Data organisation

The same data are provided in Excel and SPSS format. In addition to the information provided in Table 1, the Excel file also provides the rats' behavioural group allocation in the raw data sheet (column 4). As allocation to behavioural group conditions is balanced for each of the control groups, this variable is unlikely to show much systematic effect. We have nonetheless included it for completeness (the abbreviations used to denote the behavioural groups can be clarified by the further details provided in the corresponding publication reference). The summary sheet otherwise duplicates the information provided in Table 1.

The SPSS file includes the original rat id and an abbreviated form of the Table 1 study descriptor based on the experimental intervention (labelled cohort). The data labels in the subsequent columns refer to the brain region and neurotransmitter/metabolite sampled using the standard abbreviations defined above.

### 2 Experimental design, materials, and methods

These are fully described in the earlier published papers [[3], [4], [5], [6], [7]]. All procedures conducted prior to the post mortem tissue sampling and assays were carried out in accordance with the United Kingdom (UK) Animals Scientific Procedures Act 1986, Project Licence number: PPL 40/3163.

These previous studies followed a standard methodology for tissue sampling and HPLC-ECD. In each case, the rats were humanely killed by dislocation of the neck and decapitated; the brains were removed rapidly and dissected on a cold tray. Tissue samples were then taken using a micro-punch procedure. Using ice-chilled razor blades, three 2 mm coronal brain sections were cut. The posterior side of the slices corresponded to approximately +3, +1 and −3 mm from bregma. Subsequently, the three 2 mm coronal sections were placed posterior side up onto an ice-chilled plate. The brain regions were identified using *The Rat Brain in Stereotaxic Coordinates* [1] and sampled from the appropriate section, using 0.84 and 1.6mm punches (depending on the size of the region to be sampled). The brain samples were then immediately frozen on dry ice and stored at −80 °C until assay by HPLC-ECD to determine neurotransmitter and metabolite levels.

The data from some regions are pooled across the right and left hemispheres because the hemispheres were not separately sampled. For example, the PL and IL sub-regions of mPFC are medial to the midline and separate punches would have resulted in samples too small for assay by HPLC-ECD. Similarly (where sampled) OFC tissue was also pooled across right and left [3]. The core NAc, shell NAc, DLS and Amyg were punched by hemisphere and all the available data for these regions provided by right/left hemisphere. Thus the effect of laterality can be investigated for these regions (*N* = 100 for NAc and DLS, *N* = 53 for Amyg). However, there was no differentiation of NAc sub-regions in the study of the effects of systemic MDMA in which a larger punch was used [7]. Caudate putamen [3,5,6] and DLS [4] refer to the same sample (the same punch was used to extract the anatomically equivalent sample) and these samples are all labelled DLS in the dataset.

Just prior to HPLC-ECD, the tissue samples were homogenised in 0.1 M PCA solution by sonication and centrifuged at 17400 g for 20 min at 4 °C before amine and metabolite levels were detected using a glassy carbon electrode flow cell (VT-03 Antec) with an ISAAC reference electrode. An external standard consisting of 5-HT, DA, and metabolites, in concentrations of 10^−7^, 0.5 × 10^−7^ and 10^−8^ M was injected at a volume of 4 μl for calibration. Samples were injected onto the column at 4 μl volumes, except for PL, IL, OFC and Amyg samples which were injected at 8 μl because of the higher detection thresholds in these regions. Samples were subsequently analysed using the Alexys software data system. The Bradford assay was routinely used to adjust for protein content (using the pellet remaining after sample centrifugation).

#### 2.1 Preliminary statistical analyses

Summary data for selected regions of interest are shown in Fig. 2, Fig. 3. Fig. 2 shows how levels of DA, NA and 5-HT vary across interconnected regions of NAc and mPFC. Fig. 3 shows levels of DA, NA and 5-HT by laterality for core and shell NAc. Some variation in the available sample sizes reflects missing data in the original studies.
